# Validation of a commercially available indirect assay for SARS-CoV-2 neutralising antibodies using a pseudotyped virus assay

**DOI:** 10.1016/j.jinf.2021.03.010

**Published:** 2021-05

**Authors:** Matthew J. Murray, Megan McIntosh, Claire Atkinson, Tabitha Mahungu, Edward Wright, Wendy Chatterton, Michael Gandy, Matthew B. Reeves

**Affiliations:** a Institute of Immunity & Transplantation, University College London, Royal Free Campus, London NW3 2PF, UK; b Department of Infectious Diseases, Royal Free Hospital London NHS Foundation Trust, London NW3 2PF, UK; c School of Life Sciences, University of Sussex, Brighton, UK; d Health Services Laboratories LLP, London, UK

## Abstract

**Objectives:**

To assess whether a commercially available CE-IVD, ELISA-based surrogate neutralisation assay (cPass, Genscript) provides a genuine measure of SARS-CoV-2 neutralisation by human sera, and further to establish whether measuring responses against the RBD of S was a diagnostically useful proxy for responses against the whole S protein.

**Methods:**

Serum samples from 30 patients were assayed for anti-NP responses, for ‘neutralisation’ by the surrogate neutralisation assay and for neutralisation by SARS-CoV-2 S pseudotyped virus assays utilising two target cell lines. Correlation between assays was measured using linear regression.

**Results:**

The responses observed within the surrogate neutralisation assay demonstrated an extremely strong, highly significant positive correlation with those observed in both pseudotyped virus assays.

**Conclusions:**

The tested ELISA-based surrogate assay provides an immunologically useful measure of functional immune responses in a much quicker and highly automatable fashion. It also reinforces that detection of anti-RBD neutralising antibodies alone is a powerful measure of the capacity to neutralise viral infection.

## Highlights

• An ELISA based cPass assay provides a measure of Spike RBD binding antibodies.
• Binding in a cPass ELISA correlates strongly with pseudotyped virus neutralisation.
• ELISA-based surrogate assays provide rapid data on neutralising capacity.

## Introduction

SARS-CoV-2, the aetiological agent of COVID-19 disease, has been the focus of intense research efforts since its emergence in late 2019. Development of clinical interventions and diagnostic tools has proceeded at a rapid pace. However, as we move towards the deployment of widespread vaccination programmes, additional challenges will emerge. An important aspect moving forward will be the capacity for long term monitoring of the functional immune response against SARS-CoV-2 at a population level. Whilst SARS-CoV-2 infection is known to elicit potent neutralising antibody responses, these can wane within the span of a few months, particularly in those who only suffer a mild infection.^1^^,^^2^ However, an independent study demonstrated that whilst antibody titres may drop the specific neutralising activity of the antibody response improves between 1 and 6 months post infection. Furthermore, the authors reported stable levels of circulating memory B cells suggesting that individuals will be better protected upon re-exposure – a fundamental principle of immunological memory.^3^^,^^4^ These studies exemplify the importance of monitoring antibody responses and, furthermore, the quality of the antibody response. To date, antibody titres can be assessed by commercial assays but, often, the antibodies measured in these assays are typically those that target the nucleocapsid protein (NP), and no attempt is made to measure how functional these responses are.

A more immunologically relevant viral target for antibodies is the SARS-CoV-2 surface glycoprotein spike (S). The S protein facilitates binding to human angiotensin-converting enzyme-2 (ACE-2) via its receptor-binding domain (RBD).^5^, ^6^, ^7^, ^8^ The isolation of various highly potent monoclonal antibodies directed against the RBD reinforces the importance of this particular region of the S protein.^9^, ^10^, ^11^ Consequently, long term monitoring of specifically neutralising antibody levels against S protein, or just the RBD, is likely to provide a more clinically useful measure of functional immunity against SARS-CoV-2. This is heightened even further by the fact that vaccine development has logically focused on generating immune responses against the S protein,^12^, ^13^, ^14^, ^15^ and thus these responses would not be detected by an NP-specific assay.

Ideally, neutralising antibody responses would be assayed by measuring the ability of patient sera to prevent infection of physiologically relevant target cells (e.g. primary lung epithelial cells) by SARS-CoV-2. However, this requires a high level of expertise, equipment and containment facilities, and is not feasible on a large scale. An attractive alternative is the generation of pseudotyped viruses, often used in vitro for genetic modification of cells, which are produced from a combination of multiple plasmids and thus cannot propagate in isolation.^16^ Although this approach does have limitations it does allow specific analysis of antibody responses against S protein in a more high-throughput manner.

Whilst pseudotyped viruses represent a highly tractable middle ground between studying fully infectious SARS-CoV-2 and studying proteins in isolation, they still require a level of expertise to utilise effectively, are vulnerable to biological and experimental variation and assays that employ them can take over 24 h, potentially extending to multiple days to return results. Thus, a validated measure of neutralising antibody responses against S protein that could be measured in a simple rapid ELISA-type assay has important implications for large scale rapid assessment of antibody activity. Thus our remit was to determine whether a commercially available ELISA-type surrogate virus neutralisation kit (Genscript cPass SARS-CoV-2 Surrogate Virus Neutralization Kit), which claims to specifically measure neutralising antibodies against SARS-CoV-2 S RBD was capable of: a) detecting neutralising antibody responses in serum samples confirmed positive for antibodies against NP, b) whether those responses correlated with those determined by pseudotyped virus neutralisation assay and therefore c) whether measuring responses solely against the RBD of S protein is indicative of responses against the S protein as presented in a viral context.

During the course of our own studies, it was reported that using a similar approach, evidence of correlation between the surrogate virus neutralisation assay and neutralisation of both SARS-CoV-2 S pseudotyped viruses and wildtype SARS-CoV-2 was shown.^17^ However, we note that in the prior study that the authors used a VSV-pseudotyped virus and performed the study in vero cells. Given the increasing uncertainty around the use of vero cells as a reliable model of aspects of SARS-CoV-2 entry,^18^ we completed our own analyses with an alternative pseudotyping (lentivirus) system and, importantly, using human cells expressing TMPRSS2 as targets for infection.

## Methods

### Cell culture conditions

Hela cells constitutively expressing ACE2 (Hela-ACE2, a kind gift from James Voss^19^) and 293T/17 (ATCC CRL-11,268) cells were incubated at 37 °C at 5% CO_2_ in DMEM (Gibco) supplemented with 10% fetal calf serum and 100 U/ml penicillin/streptomycin cocktail.

### Sample acquisition and preparation

A panel of anonymous residual serum samples surplus to diagnostic requirements from the Royal Free archive (as such use of these sera is exempt from specific ethical approval) were used to undertake the neutralisation assay assessment. These samples were previously classified as positive (*n* = 15) and negative (*n* = 15) for SARS-CoV-2 nucleocapsid antibody serology using the Roche Elecsys Anti-SARS-CoV-2 Assay. Testing was performed as per manufacturer's instructions.

Samples were heat inactivated by treatment at 56 °C for 30 min prior to usage in any further assays.

### Surrogate viral neutralisation assay (SVN assay)

Serum samples were tested for neutralising activity using the SARS-CoV-2 Surrogate Virus Neutralization Test Kit (cPass Assay, Genscript) as per manufacturer's instructions. Briefly, samples and provided positive and negative controls were diluted 1:10 with provided Sample Dilution Buffer. 125 µl of sample/control was then mixed 1:1 with HRP-RBD solution and incubated at 37 °C for 30 min. 100 µl of each sample/control was added to the provided hACE2 coated plate in technical duplicate. Plate was sealed and incubated at 37 °C for 15 min. Wells were then washed 4x with 200 µl of provided Wash Solution. 100 µl provided TMB solution was added per well and the plate incubated in the dark at room temperature for 15 min. 50 ul of provided Stop Solution was added per well to quench reaction, and absorbance at 450 nm was read immediately (Thermo Scientific Multiskan FC Microplate Photometer).

Data was analysed as per manufacturer's instructions. Relative inhibition was calculated by the equation:

$$Inhibition=\left(1-\frac{OD\;value\;of\;Sample}{Mean\;OD\;value\;of\;Negative\;Control}\right)*100$$

Values ≥20 were considered positive for neutralisation (as per manufacturer's instructions), whilst those <20 were considered negative. Samples were ranked in order from highest relative inhibition to lowest.

The confidence interval for assay sensitivity was computed by the Wilson-Brown method within Graphpad Prism software.

### SARS-CoV-2 pseudotyped virus production

Solutions of the required plasmids and transfection reagents were prepared thusly: 0.6 µg of pcDNA3.1-SARS-CoV-2-S (a kind gift of Nigel Temperton, University of Kent), 0.6 µg of pCMV8.91 and 0.9 µg of pCSFLW were incubated in 50 µl OptiMEM for 5 min. 6 µl of TransIT-293 (Mirus) was added to 50 µl OptiMEM (Gibco) and incubated for 5 min. Transfection reagent and plasmid mix were then combined and mixed by inversion. Mixture was incubated at room temperature for 20 min with occasional inversion, followed by dropwise addition to 70% confluent 293T/17 cells in 1 ml DMEM (Gibco) in a 6-well plate. Four hours post addition, 1.5 ml additional DMEM was added to cells. Supernatant was harvested 48 h post transfection, spun at 500 g for 5 min to remove cell debris, and stored at −80 °C.

### Transfection of cells

5 µg of pCAGGS-ACE2 and 500 µg of pCAGGS-TMPRSS2 were incubated in 500 µl OptiMEM for 5 min. 15 µl of TransIT-293 (Mirus) was added to 500 µl OptiMEM and incubated for 5 min. Transfection reagent and plasmid mix were then combined and mixed by inversion. Mixture was incubated at room temperature for 20 min with occasional inversion, followed by dropwise addition to 60% confluent 293T/17 cells in a 100 mm dish. Cells were utilised 48 h post transfection.

### SARS-CoV-2 neutralisation assay

SARS-CoV-2 pseudotyped virus (a previously established quantity sufficient to produce 400,000 RLU in 293T/17 cells transfected with TMPRSS2 and ACE2) was treated in a total volume of 100 µl with serial dilutions of sera or media only control for 1 h at 37 °C in a 96-well plate format. Then, 2.5 × 10^4^ Hela-ACE2 cells or 2.5 × 10^4^ 293T/17 + ACE2/TMPRSS2 (in 100 µl) were added to each well.After 48 h, media was removed, cells washed with PBS and cells lysed with a 1:1 mixture of complete media and Bright-Glo luciferase reagent (Promega). After 5 min, luciferase activity was read out using a luminometer (GloMax 96 Microplate Luminometer, Promega). Virus + cells only and cells only controls were included on each plate to allow for normalisation of luminescence across multiple plates.

### Analysis of pseudotyped virus data

Data from pseudotyped virus infection of Hela-ACE2 cells was ranked based on endpoint criteria, to reflect the measurements used in the SVN cPass assay. Those samples capable of reducing luciferase activity by >95% at a higher dilution than others were ranked more highly (e.g. a sample that reduced by >95% at 1:80 was ranked higher than one that reduced by >95% at 1:40 but not at 1:80). Samples that reduced by >95% at the same dilution were ranked relative to one another based on their absolute performance at the lowest dilution at which they did not display a reduction of at least 95% (e.g. Sample A and sample B reduce by >95% at 1:20, but sample A reduces by 90% at 1:40 and sample B by 80% at 1:40. Sample A would rank higher than sample B). Samples that could reduce luciferase activity by >95% at 1:10 dilution were considered positive for neutralisation, whilst those that could not were considered negative.

Due to the increased dynamic range of the assay available in 293T/17 + ACE2/TMPRSS2 cells, performance in the pseudotyped virus neutralisation assay was assessed by multiple criteria: Half complete neutralisation dilution (ND_50_, i.e. the dilution at which the serum was capable of reduced the luciferase signal by 50% of the activity observed in the absence of serum), 90% complete neutralisation dilution (ND_90_) and maximum inhibition (i.e. level of inhibition observed in the least dilute, 1:10 condition). ND_50_ was calculated using GraphPad Prism software, and ND_90_ calculated using the resultant ND_50_ and Hill slope (H) values by the equation:

$$ND_{90}=\;ND_{50}\;{\left(\frac{10}{100-10}\right)}^{1/H}$$

Samples were then ranked for each criterion according to their absolute performance. Correlation between different sets of ranked criteria was assessed within GraphPad Prism software by simple linear regression or Spearman's correlation. Correlation between sets of raw data was performed using nonparametric Spearman's correlation within GraphPad Prism software.

## Results

**Fig. 1:**
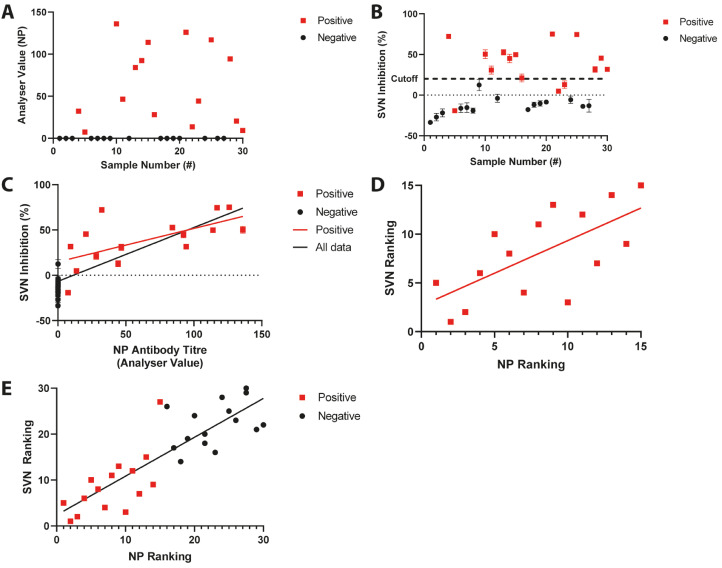
Neutralising activity in a surrogate virus neutralisation assay correlates with anti-nucleocapsid protein titre. (A)Serum samples from 30 individuals was tested for antibodies against SARS-CoV-2 nucleocapsid protein (NP) by Elecsys® Anti-SARS-CoV-2 assay. Those in red were considered positive for anti-SARS-CoV-2-NP antibodies, and are shown as such throughout the figure. (B) Serum samples from (A) were tested for neutralising activity by an ELISA-type surrogate virus neutralisation (SVN) assay. The cutoff represents the manufacturer's advised value of 20% (i.e. a 20% reduction in signal compared to the negative control). Values shown as mean ± SD. (C) Comparison between raw data from NP and SVN assays. Data was assessed by both Spearman correlation and by simple linear regression. The red line indicates the best fit linear regression line for NP-positive samples only, whilst the black line represents the best fit line for all data points. (D,E) Samples were ranked according to their NP antibody titre and neutralisation in the SVN and plotted for clarity. These ranks were then compared by simple linear regression. Data for NP-positive only samples is found in (D), whilst data for all samples is found in (E).

To perform our analyses, we first collected a bank of sera samples previously assayed for the presence of SARS-CoV-2 nucleocapsid protein (NP) reactive antibodies. Fifteen samples were confirmed to be positive for antibodies to NP, whilst the remaining 15 samples were confirmed to be negative (Fig 1A). The serostatus of the samples established by this assay was taken to be the baseline to which all following data was compared. Sera were then heat inactivated and tested in parallel in both surrogate (SVN cPass) and pseudoviral neutralisation (PVN) assays (total summary of data available in Figure S1).

The 30 samples were analysed in the SVN cPass assay, in which neutralisation is assessed by the ability of the sera to block binding of HRP-conjugated SARS-CoV-2 S receptor binding domain (RBD) to a human ACE-2-coated ELISA plate (Fig. 1B). Application of the manufacturer's advised cut-off of 20% resulted in 11 samples reporting as unambiguously positive for ‘neutralisation’, with a further sample considered ambiguously positive (technical replicates lying either side of the cut-off, but with an average neutralisation of 21%).

The remaining 18 test samples were considered negative, in addition to the provided negative control. Importantly, all 15 samples considered negative by the NP assay were also negative in the SVN cPass assay. Therefore, 3 samples considered positive by NP assay were returned as negative by the SVN assay. This could either represent false negatives or represent individuals whom failed to generate an effective neutralising response to the S protein upon SARS-CoV-2 infection. We note that two of these samples (#22 and #23) did register positive values below the manufacturer's cut-off of 20% (4.8% and 12.8%, placing them 14th and 12th in the SVN neutralisation ranking respectively), whilst all but 1 negative samples (#9 being the exception) registered negative values (i.e. most negative samples had an ELISA OD reading above that of the provided negative control). One interpretation is that the kit did detect a level of neutralising activity in these samples, but it was below the limit of sensitivity. The third ‘false negative’ (#5) performed extremely poorly, demonstrating less neutralisation than 12 negative samples. By this analysis, the SVN kit demonstrated a sensitivity of 80% (95% C.I.: 0.548–0.930) and a specificity of 100% based on NP antibody titres.

Next, we investigated whether SVN cPass assay performance correlated with responses against NP protein. Initially, we compared the raw values produced by the two assays for only those samples considered to be positive for NP reactive antibodies by two measures: by correlation and by simple linear regression (Fig 1C). This resulted in a correlation coefficient, r, of 0.668 (95% CI: 0.0.221–0.883, *p* = 0.008) and an *R*^2^ value of 0.409 (*p* = 0.0001) respectively. Ranked data was also plotted directly and tested by simple linear regression for clarity (Fig 1D, *R* = 0.668). When these analyses were extended to include those samples negative for NP-reactive antibodies (Fig 1D/E), the correlation increased to *r* = 0.848 (95% CI 0.694–0.926, *p* < 0.0001) and the *R*^2^ value for simple linear regression increased to 0.669 (*p* < 0.0001). Whilst these correlations are all highly significant, the relatively poorer performance when only considering samples positive for NP-reactive antibodies is likely indicative of the fact the assays test for different antibody functions; namely, ability to bind NP protein against claimed ability to prevent S protein RBD from binding the ACE2 receptor.

In order to assess whether the SVN cPass assay was genuinely capable of providing a reliable measure of neutralising activity, the same samples were used in two SARS-CoV-2 lentiviral pseudotyped virus neutralisation (PVN) assays, employing different target cells. Sera samples were serially diluted and mixed with pseudovirus particles bearing SARS-CoV-2 S envelope proteins, before the addition of either SARS-CoV-2 receptor ACE2-expressing Hela cells, or 293T/17 cells transfected to transiently express ACE2 and TMPRSS2. Successful entry into the cell by the virus resulted in integration of a luciferase-expressing lentivirus construct, whose activity could be read out using standard luciferase techniques. Samples were then ranked according to criteria described in the methods.

**Fig. 2:**
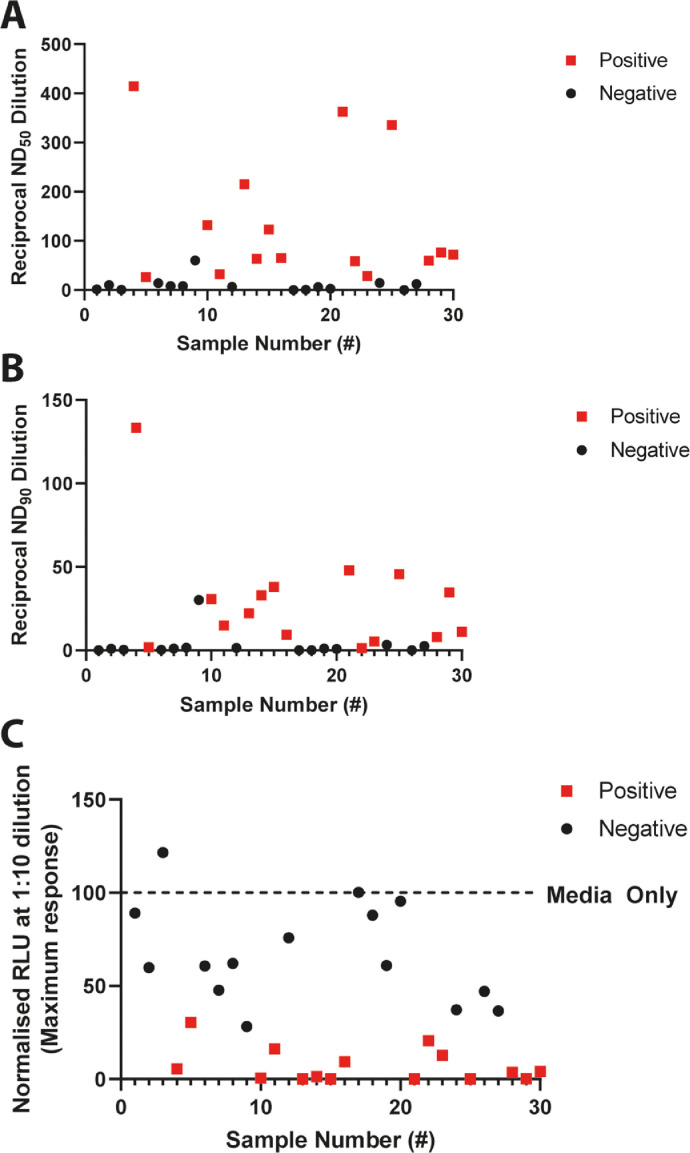
Assessment of neutralising activity of patient serum by pseudovirus assay. SARS-CoV-2 S pseudotyped virus was incubated with a series of serum dilutions for 1 h prior to infection of 293T/17 cells transiently expressing ACE2 and TMPRSS2. Forty-eight hours post infection, luciferase activity (RLU) was readout as a measure of infection. Points in red were considered positive for anti-SARS-CoV-2-NP antibodies, and are shown as such throughout the figure. (A) Summary of dilutions at which serum could reduce RLU by 50% (ND_50_) compared to untreated controls. (B) Summary of dilutions at which serum could reduce RLU by 90% (ND_90_) compared to untreated controls. (C) Normalised RLU observed for all samples at a 1:10 dilution (‘Maximum response’).

Summary data demonstrating the ND_50_, ND_90_ and maximum response data generated from the infection of ACE2/TMPRSS2-expressing 293T/17 cells can be found in Fig 2A–C. The data indicate that all samples that were positive for NP antibodies demonstrated more potent neutralisation than all but one of the negative samples (#9), all with ND_50_ values in excess of 25 (i.e. a 1:25 dilution of serum could reduce luciferase activity by at least 50%). Sample #9 demonstrated significant neutralising activity, with an ND_50_ value of 60.0 and an ND_90_ value of 30.2, scoring higher than 5 and 8 NP-positive samples by each of these measures respectively. This is the same sample in which a below cut off degree of neutralisation was observed in the SVN assay, suggesting that this sample does indeed harbour detectable neutralising capacity against S. Additionally, sample #5 performed poorly by all measures of neutralisation (ranking behind all other samples from positive individuals and sample #9 in ND_50_ and maximum response values, and also behind a ‘negative’ sample in ND_90_ rankings). The extremely poor neutralisation demonstrated by this sample in the PVN assay recapitulates what was observed in the SVN cPass assay.

**Fig. 3:**
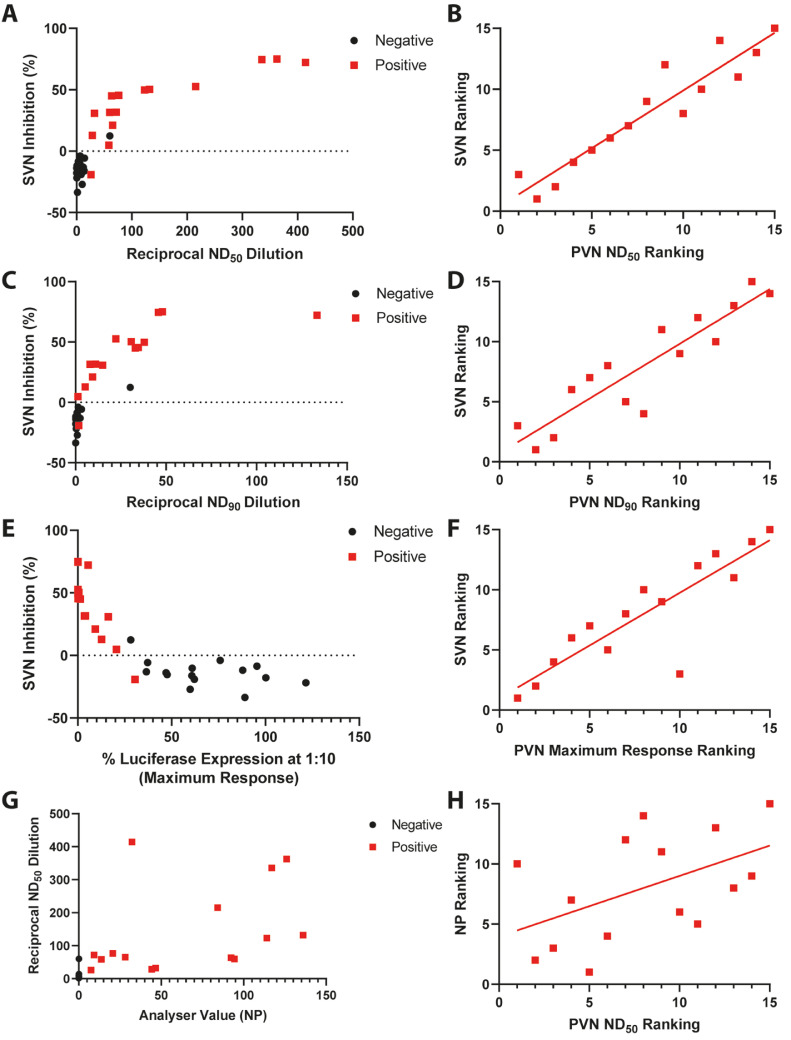
Ability of patient sera to neutralise SARS-CoV-2 pseudovirus correlates strongly with neutralisation observed in SVN assay. Data from Fig. 2 was plotted against the observed inhibition in the SVN assay and correlation assessed by Spearman correlation. Samples were then ranked according to the criteria in Fig. 2 and plotted against one another for clarity. Points in red were considered positive for anti-SARS-CoV-2-NP antibodies, and are shown as such throughout the figure. (A,B) ND_50_ values were correlated against inhibition observed in the SVN assay. (A) Represents raw values, whilst (B) demonstrates ranked values from only NP-positive samples. (C,D) ND_90_ values were correlated against inhibition observed in the SVN assay. (C) Represents raw values, whilst (D) demonstrates ranked values from only NP-positive samples. (E,F) Maximum Response values were correlated against inhibition observed in the SVN assay. (E) Represents raw values, whilst (F) demonstrates ranked values from only NP-positive samples. (G,H) ND_50_ values were correlation against anti-NP antibody titre. (G) represents raw values, whilst (H) represents ranked values from only NP-positive samples.

To judge the performance of the SVN cPass assay against the PVN assay, the performances of the samples were compared by Spearman correlation and plotted in both raw and ranked form (Fig 3). Comparison between inhibition in the SVN cPass assay and reciprocal ND_50_ values (Fig 3A/B) by only NP-reactive samples resulted in a Spearman correlation coefficient of *r* = 0.946 (95% CI 0.838–0.983, *p* < 0.0001). Comparison between SVN cPass assay and reciprocal ND_90_ values for NP-reactive samples (Fig 3C/D) produced a similarly strong correlation (*r* = 0.911, 95% CI 0.739–0.971, *p* < 0.0001), Even when employing a very blunt measure of performance within the PVN assay, namely the maximum response, correlation was extremely strong for these samples (*r* = −0.872, 95% CI −0.958 to −0.641, *p* < 0.0001). This strongly suggests that the SVN does provide a genuine measure of neutralisation against the S protein. Expanding these analyses to include samples negative for NP-reactive antibodies (Ranked plots in Fig S1A-C) results in only a small change in correlation (ND_50_, *r* = 0.871, 95% CI 0.739–0.939, *p* < 0.0001, ND_90_ *r* = 0.874, 95% CI 0.746–0.940, *p* < 0.0001 and maximum response *r* = 0.874, 95% CI, *p* < 0.0001). We also observed that when limited to positive samples only (Fig 3G/H), PVN ND_50_ values did not significantly positively correlate with anti-NP antibody titre (*r* = 0.504, 95% CI −0.028 to 0.813, *p* = 0.058). Whilst inclusion of the negative samples (Figs. 3G and S1D) led to a highly significant correlation (*r* = 0.847, 95% CI 0.694–0.926, *p* < 0.0001), this relatively poor correlation reinforces the notion that these approaches are designed to detect different aspects of the antibody response to SARS-CoV-2 infection.

**Fig. 4:**
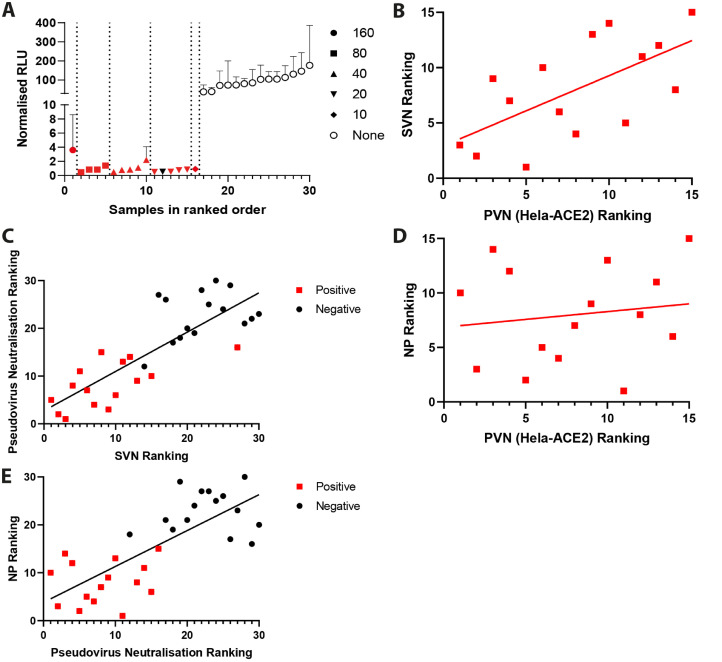
Surrogate neutralisation reported in SVN assay correlates with ability to prevent infection of Hela cells stably expressing ACE2. (A) SARS-CoV-2 S pseudotyped virus was incubated with a series of serum dilutions for 1 h prior to infection of Hela-ACE2 cells. Forty-eight hours post infection, luciferase activity (RLU) was readout as a measure of infection. Data shown indicates the RLU (compared to 100 for untreated pseudovirus) at the lowest serum dilution at which RLU was reduced by at least 95% (i.e. below 5). (B) Samples from NP-positive only donors or (C) samples from all donors were ranked as described based on data from and correlated with ranked performance in SVN by simple linear regression. (D,E) PVN ranking was compared to NP titre ranking for NP-positive only donors (D) or all donors (E). Points in red were considered positive for anti-SARS-CoV-2-NP antibodies, and are shown as such throughout the figure.

Finally, to further interrogate the suitability of the SVN assay as a surrogate measure of antibody neutralising activity in sera, we repeated the PVN in Hela cells transduced to stably express ACE2. This approach allowed us to characterise the SVN assay to an independent cell line, with lower ACE2 expression and without TMPRSS2 overexpression. Luciferase activity was much lower following infection of Hela-ACE2 cells in comparison to the transfected 293T/17 cells, and consequentially increased noise in the data rendered ND_50_ values a poor method of ranking the data. However, clear neutralisation could still be observed, and so samples were therefore ranked according to the dilution at which they could no longer reduce luciferase expression by at least 95% (Fig 4A, see Methods). By this approach, all samples that were positive for NP antibodies were positive for neutralisation (could reduce luciferase activity by 95% at a 1:10 dilution or higher), as well as one negative sample (#9, the same sample that demonstrated neutralising activity in the 293T/17 PVN assay and limited neutralisation in the SVN assay). The best performing negative sample only demonstrated a 61.7% reduction in luciferase activity at a 1:10 dilution. These rankings were once again correlated against the rankings from the SVN cPass assay, demonstrating a positive correlation coefficient of 0.636 (95% CI 0.167–0.870, *p* = 0.0128) for positive samples only (Fig 4B), which increased to 0.822 (95% CI 0.650–0.914, *p* < 0.0001) when expanded to include all samples (Fig 4C). Thus, regardless of the cell type used for the PVN assay, the readout of neutralisation activity through both approaches was highly correlative. We noted particularly poor correlation between NP-reactive antibody titre rankings and rankings in the Hela-ACE2 PVN assay for NP-positive samples (*r* = 0.143, 95% CI −0.413 to 0.621) (Fig 4D), which was again increased by the inclusion of negative samples as observed previously (*r* = 0.755, 95% CI 0.534–0.879).

## Discussion

Given the level of diagnostic activity surrounding the study of samples from COVID-19 patients, there is a pressing need for easily employable, automatable assays that go further than simply measuring total antibody titres against particular antigens. Whilst these can provide useful information, they do not constitute functional readouts of antibody activity. However, assays that can measure antibody functions such as neutralisation or capacity to trigger antibody-dependant cellular cytotoxicity (ADCC) typically involve a need for cell culture and associated biological procedures that are no longer routine in a diagnostic setting.^20^^,^^21^ Here we have demonstrated that a commercially available surrogate virus neutralisation assay produces data that strongly correlates with data from pseudotyped virus neutralisation assays, that itself has been shown to strongly correlate with wild-type, authentic SARS-CoV-2 neutralisation assays, and consequently could be employed for mass screening of individuals’ sera to measure the prevalence and intensity of neutralising antibody responses in a high-throughput manner against an important vaccine target – the S protein of SARS-CoV-2.

The ability to screen large numbers of sera samples is likely to become increasingly useful as mass vaccination programmes begin to be rolled out worldwide – particularly if the virus becomes endemic requiring seasonal vaccination. Thus, assays such as this provide an opportunity for population-level monitoring of the neutralising antibody response present in these vaccinated individuals over time and may help to establish the requirement for additional doses of the vaccine at later time points – particularly in vulnerable patient groups. Whilst neutralising antibody responses are not the only immunologically relevant measure of vaccine efficacy, they are more readily measurable in large quantities of samples in comparison to measurements of e.g. virus-specific CD4+ or CD8+ cells.^22^, ^23^, ^24^ Furthermore, assays such as this are far more relevant in a vaccination setting than the currently employed anti-NP approach, as most vaccines in development are designed to elicit protective immune responses against the S protein.^15^ It is worth noting that the accuracy of this assay would require monitoring in the context of emerging variants, particularly those with multiple mutations in the RBD.^25^, ^26^, ^27^ However, the simplicity of the assay should render testing of multiple RBD-HRP constructs, if required, readily achievable.

An additional insight from our study is comparison of how strongly neutralising responses against the whole S protein presented in the context of a virus particle correlate with responses against only the RBD. This is promising for the development of further diagnostic tools to measure relevant responses against the S protein, as use of only the RBD may be required, simplifying the production process and removing the need to produce stable forms of full length S protein in the absence of a lipid membrane to embed into.

However, we note that a previous study published during the course of our analyses suggested that the correlation between the two approaches was low.^17^ Reasons for the difference are unclear. Although a different pseudotyped virus backbone was used there is no obvious technical reason why differences would occur. A major difference was the use of Vero cells in the prior study rather than the human ACE-2 transfected cells used here. Entry into Vero cells is clearly sensitive to neutralising antibodies, but the precise mechanisms differ – a difference that led to the aberrant espousal of hydroxychloroquine as an antiviral.^18^^,^^28^ Essentially in our system Spike is processed at the plasma membrane which may make it more susceptible to antibody recognition and more likely reflects the route of natural infection.

A second point of caution (based on our analyses) over the surrogate virus neutralisation assay was the observed sensitivity of 80% which may actually be too stringent for characterising serostatus. However, optimisation of the process and, in particular, of the cut-off value (generated via the value of the negative control) would likely improve this and can be incorporated into future assay standardisation and quality control. Additionally, all samples were subjected to heat inactivation prior to use to remove components of the complement system for the PV neutralisation assay. Although we analysed the same sera in both assays this is not required for the SVN cPass assay and may have led to a minor level of antibody degradation, thus contributing to this reduced sensitivity. Whilst the kit currently does not claim to be suitable for quantitative analysis of neutralising responses, the strong correlations observed between performance in SVN and PVN suggest that it could be utilised for this purpose – if used alongside appropriate standards. In vivo there are always degrees of neutralising responses to target proteins, even in the absence of cognate infection, and evidence of a weak level of neutralisation does not indicate evidence of effective neutralisation, thus employing these tools in a quantitative manner is likely to be more informative (particularly if longitudinal samples from individuals are taken, e.g. to monitor for decline in responses following vaccination) than applying a simple yes/no cut-off.

Although the focus of our study was to validate the SVN cPass assay we were intrigued by the identification of an individual with apparent neutralising activity against S protein (albeit quite low) which was considered negative by nucleocapsid assays. Whether this reflects a more potent response against S protein in that individual or, possibly, the presence of cross-reactive antibodies against the spike proteins of circulating seasonal coronaviruses is not clear.^29^^,^^30^ However, the SVN cPass assay is focused on the RBD which, in analyses thus far, has not been identified as a major target of cross-neutralising antibodies and thus we cannot rule out a non-specific serum effect active in this sample. Additionally, the nature of the sampling means we cannot associate samples with time of infection, and it has been shown that a positive test by Roche assay is more robust later into the infection course than Spike serology. Unfortunately, the anonymisation of the samples means that this individual cannot be followed up with further tests to see if they become positive on the Roche assay.

The inherent nature of the samples (anonymised residual diagnostic samples) is a source of limitation to the study. Little is known about the disease status and the proportion of which are from otherwise healthy asymptomatics – information that cannot be identified retrospectively in this study. Furthermore, there is no direct testing against live virus on primary cells although the pseudotype approach as a surrogate has been extensively characterised for this and other infections.^31^^,^^32^

In summary, ELISA-type surrogate virus neutralisation assays, such as the Genscript cPass assay evaluated here, have the potential to reflect physiologically relevant neutralisation of SARS-CoV-2 using human cells expressing ACE-2 and TMPRSS2. Consequently, their ability to be automated and performed rapidly renders them a highly potent diagnostic tool for the ongoing monitoring of functional immune responses against the pandemic virus, at both the individual and particularly at the population level.

## Declaration of Competing Interest

None.

## Appendix Supplementary materials

Image, application 1

Image, application 2

## References

[1] Jeffrey S., Carl G., Blair M., Sam A., Suzanne P., Steel Kathryn J.A. (2020). Longitudinal observation and decline of neutralizing antibody responses in the three months following SARS-CoV-2 infection in humans. Nat Microbiol.

[2] Callow K.A., Parry H.F., Sergeant M., Tyrrell D.A.J (1990). The time course of the immune response to experimental coronavirus infection of man. Epidemiol Infect.

[3] Rodda Lauren B., Jason N., Laila S., Pruner Kurt B., Morawski Peter A., Thouvenel Christopher D. (2021). Functional SARS-CoV-2-specific immune memory persists after mild COVID-19. Cell.

[4] Christian G., Zijun W., Lorenzi Julio C C., Frauke M., Shlomo F., Minami T. (2020). Evolution of antibody immunity to SARS-CoV-2. BioRxiv Prepr Serv Biol.

[5] Jun L., Jiwan Ge, Jinfang Yu, Sisi S., Huan Z., Shilong F. (2020). Structure of the SARS-CoV-2 spike receptor-binding domain bound to the ACE2 receptor. Nature.

[6] Jian S., Gang Ye, Ke S., Yushun W., Chuming L., Hideki A. (2020). Structural basis of receptor recognition by SARS-CoV-2. Nature.

[7] Michael L., Andrea M., Vincent M. (2020). Functional assessment of cell entry and receptor usage for SARS-CoV-2 and other lineage B betacoronaviruses. Nat Microbiol.

[8] Markus H., Hannah K.-.W., Simon S., Nadine K., Tanja H., Sandra E. (2020). SARS-CoV-2 cell entry depends on ACE2 and TMPRSS2 and is blocked by a clinically proven protease inhibitor. Cell.

[9] Xiaolong T., Cheng Li, Ailing H., Shuai X., Sicong Lu, Zhengli S. (2020). Potent binding of 2019 novel coronavirus spike protein by a SARS coronavirus-specific human monoclonal antibody. BioRxiv.

[10] Kaewta R., Balamurugan S., Suwimon M., Budi P.P., Konlavat S., Narach K. (2020). Rapid production of SARS-CoV-2 receptor binding domain (RBD) and spike specific monoclonal antibody CR3022 in Nicotiana benthamiana. Sci Rep.

[11] Tal N.-.P., Efi M., Ron A., Adva M., Yinon L., Adi B.-.K. (2020). A panel of human neutralizing mAbs targeting SARS-CoV-2 spike at multiple epitopes. Nat Commun.

[12] Polack Fernando P., Thomas Stephen J., Nicholas K., Judith A., Alejandra G., Stephen L. (2020). Safety and Efficacy of the BNT162b2 mRNA Covid-19 Vaccine. N Engl J Med.

[13] Merryn V., Costa C.S.A., Madhi Shabir A., Weckx Lily Y., Folegatti Pedro M., Aley Parvinder K. (2020). Safety and efficacy of the ChAdOx1 nCoV-19 vaccine (AZD1222) against SARS-CoV-2: an interim analysis of four randomised controlled trials in Brazil, South Africa, and the UK. Lancet.

[14] Baden Lindsey R., El Sahly Hana M., Brandon E., Karen K., Sharon F., Rick N. (2020). Efficacy and Safety of the mRNA-1273 SARS-CoV-2 Vaccine. N Engl J Med.

[15] Florian K. (2020). SARS-CoV-2 vaccines in development. Nature.

[16] Jianhui N., Qianqian Li, Jiajing Wu, Chenyan Z., Huan H., Huan L. (2020). Establishment and validation of a pseudovirus neutralization assay for SARS-CoV-2. Emerg Microbes Infect.

[17] M. Benjamin, R. Johan, T. Giulia, B. Fion, Y. Sabine, H. Marieke, et al. Validation and clinical evaluation of a SARS- CoV-2 surrogate virus neutralisation test (sVNT) 2020. doi: 10.1080/22221751.2020.1835448.

[18] Markus H., Kirstin M., Heike H.-., Artur K., Hannah K.-., Nadine K. (2020). Chloroquine does not inhibit infection of human lung cells with SARS-CoV-2. Nature.

[19] Rogers Thomas F., Fangzhu Z., Deli H., Nathan B., Alison B., Wan-Ting He (2020). Isolation of potent SARS-CoV-2 neutralizing antibodies and protection from disease in a small animal model. Science.

[20] Alfred S., Roger G., Peter S., Thomas S., Stefan M., Marcel Z. (2011). Development of a quantitative, cell-line based assay to measure ADCC activity mediated by therapeutic antibodies. Mol Immunol.

[21] Lewis George K., Ackerman Margaret E., Gabriella S., Christiane M., Marjorie R.-.G., Kent Stephen J. (2019). Knowns and unknowns of assaying antibody-dependent cell-mediated cytotoxicity against HIV-1. Front Immunol.

[22] Le Bert Nina T.A.T., Kamini K., Tham Christine Y.L., Morteza H., Adeline C. (2020). SARS-CoV-2-specific T cell immunity in cases of COVID-19 and SARS, and uninfected controls. Nature.

[23] Takuya S., André P.-.P., Olga R.-.B., Kristoffer S., Baptiste G.J., Annika O. (2020). Robust T cell immunity in convalescent individuals with asymptomatic or mild COVID-19. Cell.

[24] Arne S., Stefan A., Helena S., Moira H.K., Dmytro K., Sascha T. (2020). SARS-CoV-2-specific T cell responses and correlations with COVID-19 patient predisposition. J Clin Investig.

[25] Volz Erik M., Swapnil C., Meera B., Jeffrey C., Robert J., Susan H. (2021). Transmission of SARS-CoV-2 lineage B.1.1.7 in England: insights from linking epidemiological and genetic data. MedRxiv.

[26] Houriiyah T., Eduan W., Marta G., Arash I., Vagner F., Jennifer G. (2020). Emergence and rapid spread of a new severe acute respiratory syndrome-related coronavirus 2 (SARS-CoV-2) lineage with multiple spike mutations in South Africa. Preprints.

[27] Voloch Carolina M., da S.F.R., de Almeida Luiz G P., Cardoso Cynthia C., Brustolini Otavio J., Gerber Alexandra L. (2020). Genomic characterization of a novel SARS-CoV-2 lineage from Rio de Janeiro, Brazil.. MedRxiv.

[28] Manli W., Ruiyuan C., Leike Z., Xinglou Y., Jia L., Mingyue Xu (2020). Remdesivir and chloroquine effectively inhibit the recently emerged novel coronavirus (2019-nCoV) in vitro. Cell Res.

[29] Ng Kevin W., Faulkner Nikhil C., Georgina H., Annachiara R., Ruth H., Saira H. (2020). Preexisting and de novo humoral immunity to SARS-CoV-2 in humans. Science.

[30] Aldridge Robert W., Dan L., Sarah B., Johnson Anne M., Maria Z., Hayward Andrew C. (2020). Seasonality and immunity to laboratory-confirmed seasonal coronaviruses (HCoV-NL63, HCoV-OC43, and HCoV-229E): results from the Flu Watch cohort study. Wellcome Open Res.

[31] Brett C.J., W. R.P., Chen Rita E., Zhuoming L., Haiyan Z., Kim Arthur S. (2020). Neutralizing antibody and soluble ACE2 inhibition of a replication-competent VSV-SARS-CoV-2 and a clinical isolate of SARS-CoV-2. Cell Host Microbe.

[32] Schmidt F., Weisblum Y., Muecksch F., Hoffmann H.-.H., Michailidis E., Lorenzi J.C.C. (2020). Measuring SARS-CoV-2 neutralizing antibody activity using pseudotyped and chimeric viruses. J Exp Med.

