# Supplementary material for: Validation of a commercially available indirect assay for SARS-CoV-2 neutralising antibodies using a pseudotyped virus assay
Source: J Infect. 2021 May;82(5):170–7. doi: 10.1016/j.jinf.2021.03.010 (PMC7979278; doi:10.1016/j.jinf.2021.03.010)

**Supplementary Figure 1: Ability of patient sera to neutralise SARS-CoV-2 pseudovirus correlates strongly with neutralisation observed in SVN assay.**

All samples were ranked based on the criteria indicated in Figure 2, and these rankings correlated against ranked performance in the SVN assay. Points in red were considered positive for anti-SARS-CoV-2-NP antibodies, and are shown as such throughout the figure. (A) Rankings based on ND_50_ were correlated against rankings from SVN. (B) Rankings based on ND_90_ were correlated against rankings from SVN. (C) Rankings based on ‘Maximum response’ were correlated against rankings from SVN. (D) Rankings based on ND­_50_ were correlated against NP titre ranking. Correlation was determined by simple linear regression in all panels. Data is associated with Figure 3.


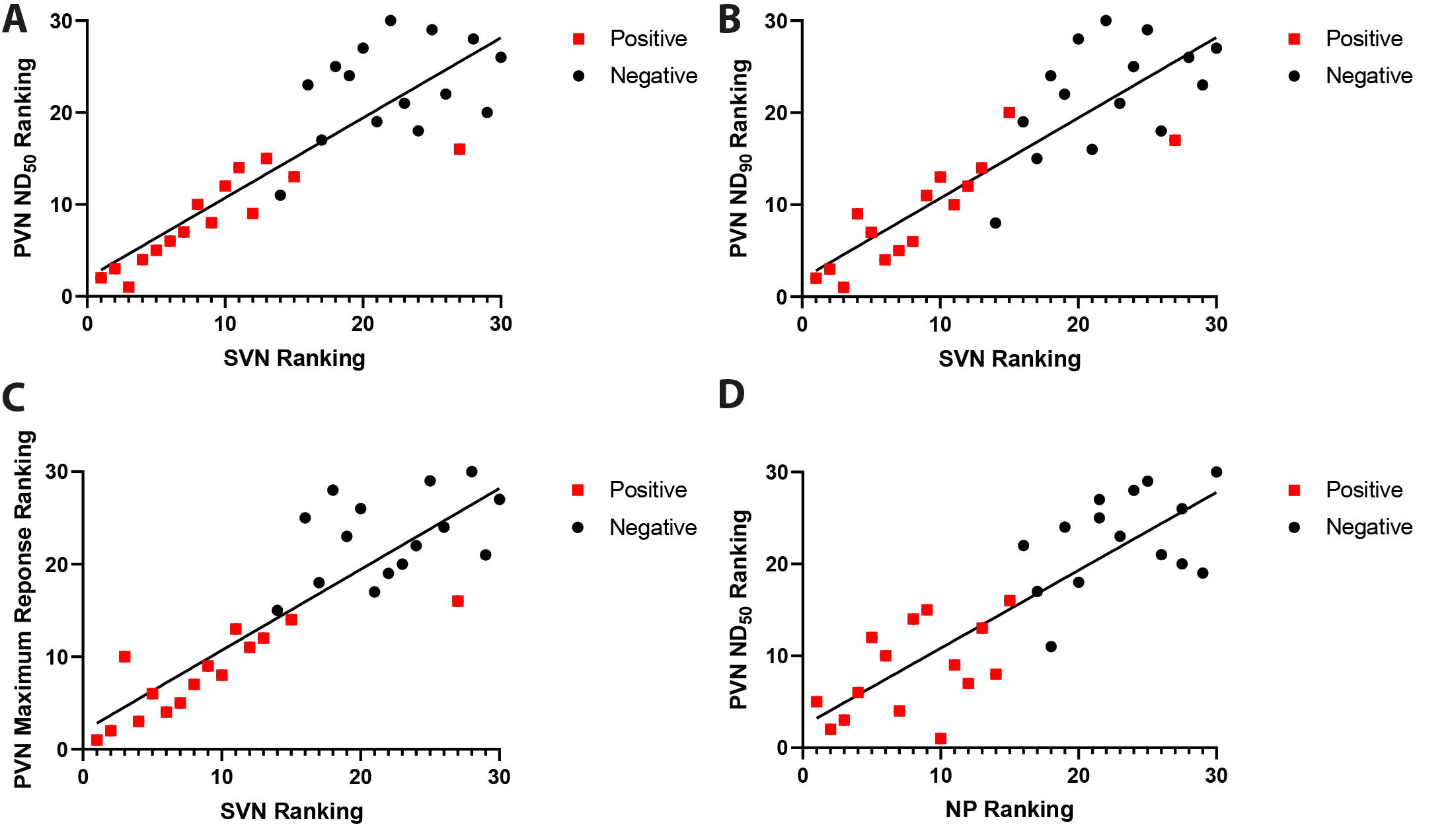

Supplement: Supplementary file 1 [file mmc1.docx]
